# Biomechanical investigation of the supraorbital arch - a transient FEA study on the impact of physical blows

**DOI:** 10.1186/1746-160X-10-13

**Published:** 2014-04-21

**Authors:** Heike Huempfner-Hierl, Andreas Schaller, Thomas Hierl

**Affiliations:** 1 Department of Oral & Maxillofacial Plastic Surgery, Leipzig University, Liebigstr. 10-14, 04103 Leipzig, Germany; 2 BBG Bodenbearbeitungsgeraete Leipzig GmbH & Co KG, Leipzig, Germany

**Keywords:** Supraorbital fracture, FEA-simulation, Craniomaxillofacial trauma

## Abstract

**Introduction:**

As fractures of the supraorbital region are far less common than midfacial or orbital fractures, a study was initiated to investigate whether fist blows could lead to fractures similar to those often seen in the midface.

**Methods:**

A detailed skull model and an impactor resembling a fist were created and a fist blow to the supraorbital region was simulated. A transient finite element analysis was carried out to calculate von Mises stresses, peak force, and impact time.

**Results:**

Within the contact zone of skull and impactor critical stress values could be seen which lay at the lower yield border for potential fractures. A second much lower stress zone was depicted in the anterior-medial orbital roof.

**Conclusions:**

In this simulation a fist punch, which could generate distinct fractures in the midface and naso-ethmoid-orbital region, would only reach the limits of a small fracture in the supraorbital region. The reason is seen in the strong bony architecture. Much higher forces are needed to create severe trauma in the upper face which is supported by clinical findings. Finite element analysis is the method of choice to investigate the impact of trauma on the human skeleton.

## Background

Fractures in the supraorbital region represent only a small group within craniomaxillofacial trauma [1-3] and are related to high-impact trauma or complex fractures of the skull and midface. As trauma of the supraorbital region may lead to vision threatening orbital blow-in fractures, it was of interest to investigate whether impacts (i.e. fist punches) that are a main cause for zygomatic, infraorbital and orbital wall fractures could also cause similar dislocation patterns in the supraorbital arch. Biomechanical investigations utilizing the finite element analysis (FEA) have been proven efficient in the mandible and midface [4-7] and were therefore chosen for this investigation.

The supraorbital arch forms the superior enclosure of the orbit and shows an interesting different architecture than the infraorbital bony structures. It consists of two convex chords in the frontal sinus region connected by bony struts. This resembles principles used to build durable constructions like arch dams or arch bridges which utilize less material to realize high mechanical needs. From a biomechanical viewpoint this suggests a higher resistance to blunt forces. As no studies on this topic are known to the authors, the following investigation was set up.

## Methods

**Figure 1:**
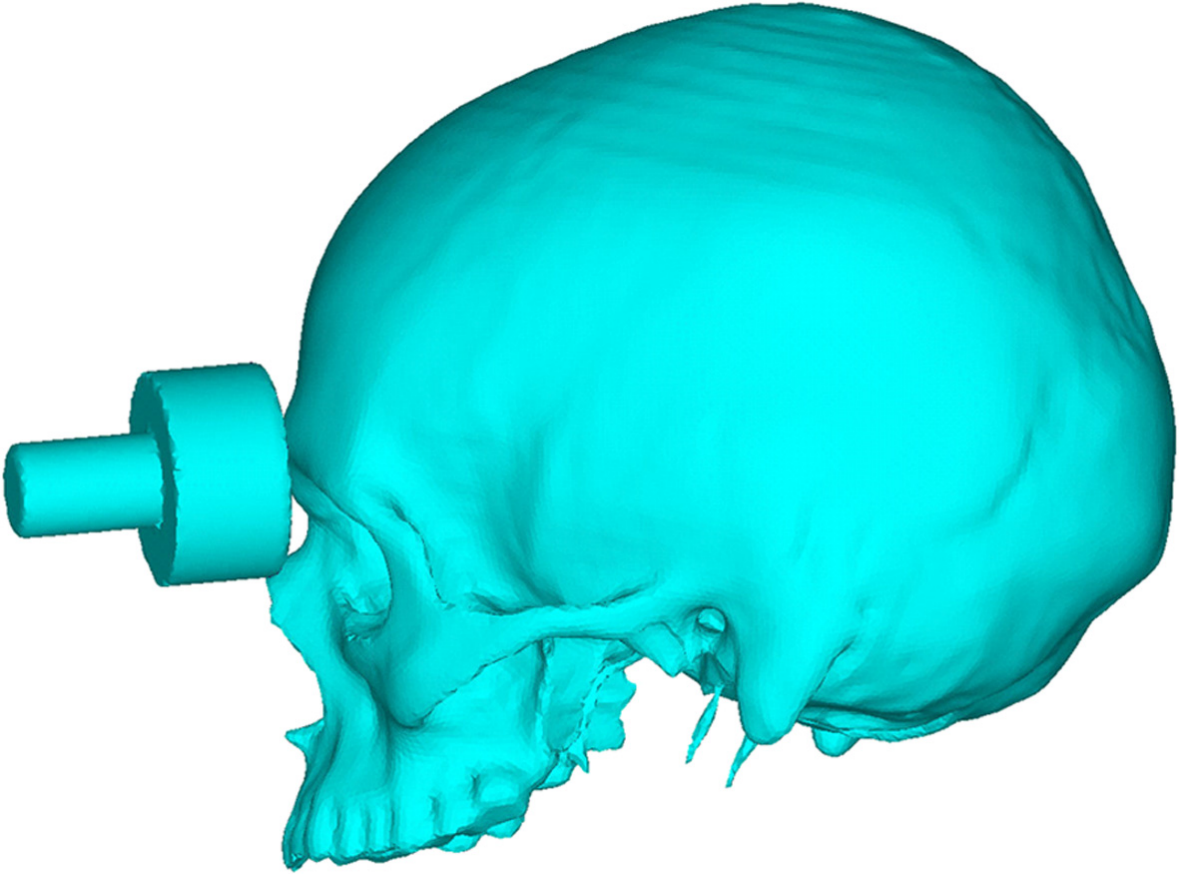
**Impactor and skull model seen from lateral view.** The contact point lies in the medial aspect of the supraorbital rim.

To simulate the effects of a fist blow on the supraorbital arch a detailed finite element model was built from a CT dataset of a young man without any pathological findings. Then an impactor resembling a fist was constructed according to suggestions of Waterhouse et al. [8] and directed to the supraorbital rim (Figure 1). A transient simulation was now carried out to analyze the dynamic response under the time dependant load.

The detailed setup of the FEA-simulation and model generation has been given before [4,5], therefore only a shortened description is stated.

A clinical CT dataset consisting of 1 mm contiguous slices resembling a typical trauma patient (male, 34 years, no concomitant pathological findings) was used. To transfer a patient CT into a realistic FEA model, two major steps must be considered. First scanning resolution must allow detection of the relevant bony structures and secondly, segmentation should lead to a model with correct anatomical structures (i.e. no non-existing holes or masses) in the correct dimension. For this study resolution in the axial XY-plane lay at 0.3-0.4 mm at the chosen field of view. Regarding Z-axis a resolution of about 0.8 mm resulted. As automatic segmentation based on Hounsfield value thresholds fails in the intricate thin cortical bone, the following approach was chosen: after presegmentation, each slice was edited manually in axial, sagittal, and coronal reformation. Based on visual detectable differences between air/soft tissue and grey values representing bone as well as orientating on the neighbouring bone structures, a one-pixel brush (width 0.3 mm) was applied to close all gaps and add bone in the appropriate thickness. Thus a contiguous model sparing only known anatomical foramina was created (VWorks 4.0; Cybermed Co. Seoul, Korea). In a next step the model was smoothed and exported as a VRML file omitting the mandible. Then the file was imported into ANSYS ICEM CFD 12.01 (ANSYS Inc., Canonsburg, PA, USA) (ICEM). Next a volume mesh consisting of about 740 000 tetrahedral-shaped 10-node-elements was created. An impactor of 0.049 cm^3^ volume consisting of two cylinders, the larger one with a diameter of 45 mm and thickness of 25 mm was constructed according to literature [8-11]. Now all ICEM files were transferred to ANSYS Classic v12.0.1 (ANSYS Inc.) for the transient nonlinear solution. In contrast to static FEA investigations, transient stands for the assumption that the impactor-bone interaction and the applied force are time dependent. This implies that the exerted force leads to a gradient excitation like an oscillation which could be a more realistic setting in trauma studies. In this investigation the explicit method for solving was chosen as this is the method of choice for fast phenomena.

**Figure 2:**
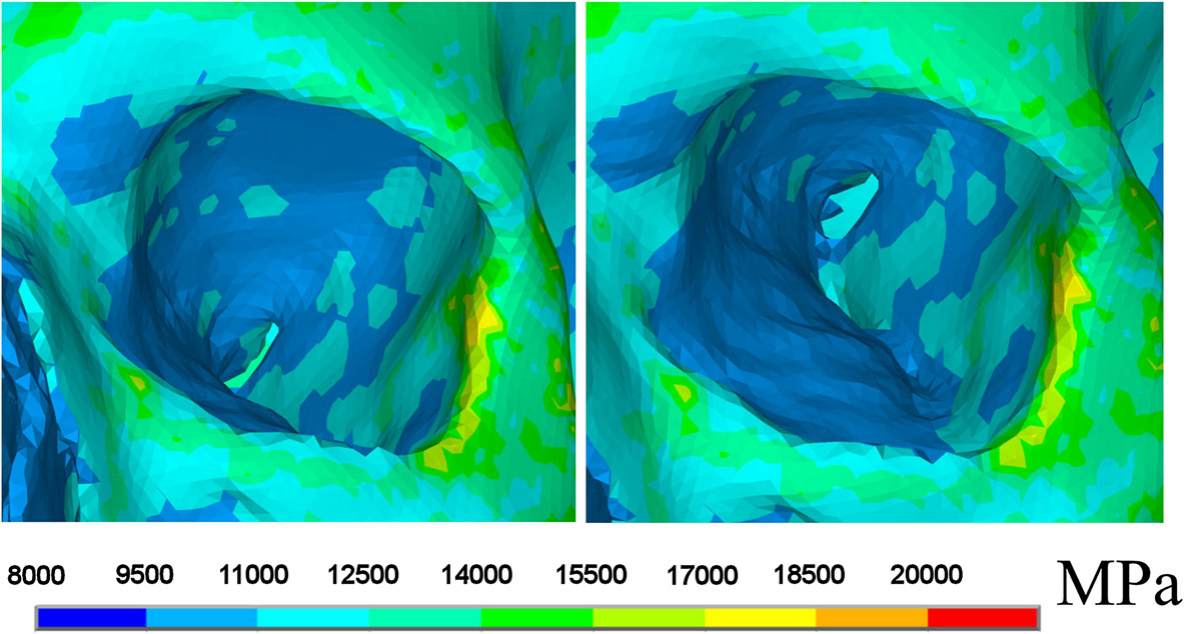
**Colour-coded assignment of mechanical bone property parameters of the orbit and the periorbital region in inferior and superior oblique views.** Young’s moduli of each element are given in megapascal (MPa).

For a most realistic simulation, no standard material values for the skull were chosen. Instead, individual Young’s modules were calculated for all single elements using the software programme BoneMat^©^[12,13]. First each CT pixel was attributed the individual Hounsfield value. Next, density values were calculated and the Young’s modules were created by a density approach [12,14-16]. Thus each volume mesh element was assigned with individual material parameters (Figure 2). In addition, a Poisson ratio of 0.326 was chosen [17]. To achieve a realistic numeric calculation of stresses, the Young’s modulus of the model was reduced by confining the lowest value to 11 000 MPa [10,11,18]. For the impactor a density of 8.4 g/cm^3^, a Poisson ratio of 0.37, and a Young’s modulus of 100 000 MPA were utilized.

Regarding boundary conditions, the upper occipital bone was fixed in all degrees of freedom. The impactor hit the skull with 6 m/s equalling fist punches [19] (Figure 1). A Coulomb friction model with a 0.3 coefficient was set up for the contact pair of skull and impactor [20].

To evaluate the results the von Mises stresses were examined. Here a yield criterion of the skull bone of 150 MPa was used [11]. Stresses above that level would cause destruction, which would be shown by a change from elastic to plastic deformation. According to mechanical engineering, the examined part fails if two fronts of stress gradients above the yield limit meet. Then the part is unable to resist the given load and breaks.

According to the local institutional review board no ethical approval was required for this study.

## Results

**Figure 3:**
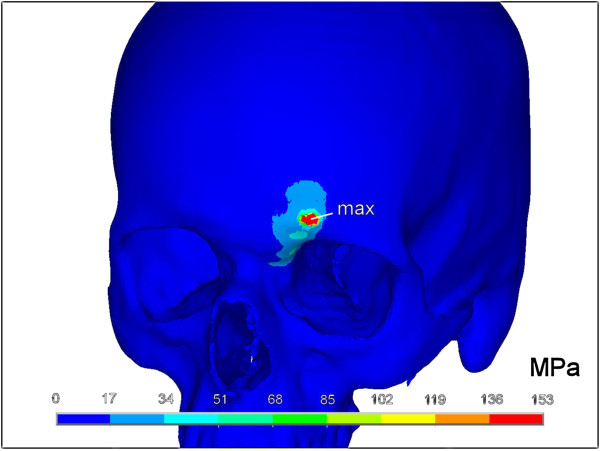
**Fracture pattern seen from left anterior-superior viewpoint.** A small punctual area reaches the yield criterion for a possible fracture (depicted in red and labelled max). The colour-coded bar states Von Mises stresses in megapascal (MPa).

**Figure 4:**
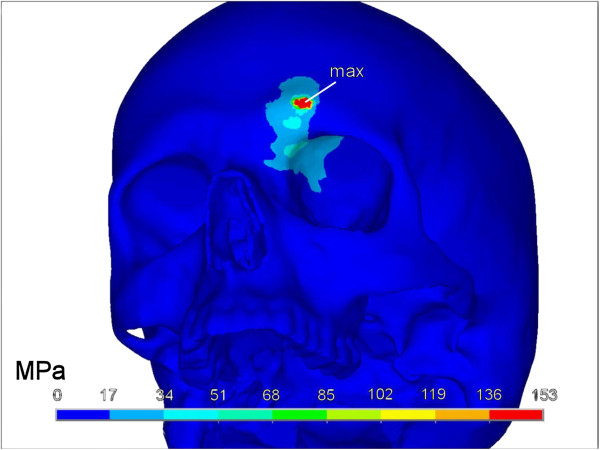
**Fracture pattern seen from an oblique inferior aspect.** Besides the possible fracture region (red) further areas of stress are visible in the orbital roof without reaching the yield criteria for bone failure (light blue resp. light gray). The colour-coded bar states Von Mises stresses in megapascal (MPa).

The above described method led to a highly detailed and dense skull volume mesh of 736 934 elements. Using an explicit transient FE analysis, von Mises stresses, peak impact force and impact time interval were calculated. Thus a total impact force of 3900 N during a time increment of 1.1 ms could be seen. The results of the transient analysis of the simulated fist blow are depicted in Figures 3 and 4.

With an assumed von Mises yield criterion of 150 MPa, a small punctual area around the contact zone in the outer table of the frontal sinus was noticed which reached the limits of possible bone failure. Looking at von Mises stresses, two areas could be discerned. First the contact zone with highest values at the contact point and secondly the anterior-medial part of the orbital roof. Here, however, the impact of a simulated fist blow did not reach critical values. While the contact area between bone and impactor measured 40 mm^2^, the area showing highest von Mises stresses was 22 mm^2^ in the outer cortex of the frontal bone.

## Discussion

The discussion of this investigation can be viewed under two aspects. First, how valid is the chosen model and second, what are the clinical results.

Regarding the simulation set-up, it can be concluded that a more realistic model should be more appropriate. Thus more elements and individual material parameters are superior to models with less elements and uniform material characteristics. Therefore the model seen here is similar to the one suggested by Szwedowski et al. [13] (740 000 elements vs 850 000) who also used BoneMat® software to attribute density related biomechanical parameters. It is superior to the one used by Nagasao et al. [11] (188 – 248 000 elements) or Gautam et al. [21] (108 799 elements) with consistent material values. It could be debated whether the inclusion of the soft tissue overlying the supraorbital region could add more realism, but as the soft tissue here – in contrast to the cheek - is rather thin and incompressible, the effect should be rather marginal. A CT-dataset of 1 mm slicing can be regarded as anatomically sufficient for model generation of the supraorbital region as presented by the authors as resolution in the axial plane lay at 0.3-0.4 mm and the manual slice editing allowed generation of cortical thin structures of 0.3 mm. In different regions other ways to generate an anatomically appropriate model could be discussed. It is obvious that higher resolution scanning, which is technically possible like 0.3 mm or less would allow superior segmentation of the thin cortical bone structures of the orbital walls. The extent of improvement concerning a superior model and more realistic results can only be supposed. As the authors wanted to model the investigation on a typical living subject were radiation exposition concerns prohibited higher exposure they did not turn to high resolution scans of skulls of senior age from the anatomy department.

In this study failure was not incorporated. Adding failure would mean that failing elements would be deleted and the simulation would have to be recalculated with this new boundary condition until further failing elements would be omitted etc. This could add information in crack propagation in brittle materials and could be investigated in future studies.

The chosen transient structural analysis allows an investigation under a time dependant load which equals the situation of real trauma. In summary, the model and simulation can be regarded appropriate for the chosen clinical questions.

Seen from a clinical viewpoint, the supraorbital rim and forehead is more stable in biomechanical terms than the midface. Low-velocity fist punches which would cause distinct fractures of the zygoma or naso-orbital-ethmoid [4,5] just reached the lower limits of potential isolated fractures, i.e. could cause fractures or not.

The resulting impact force of 3.9 kN corroborates this assumption as tests on frontal bone failure using cadaver specimens have stated impact forces between 3 – 10 kN necessary to create visible fractures in this region [22,23].

**Figure 5:**
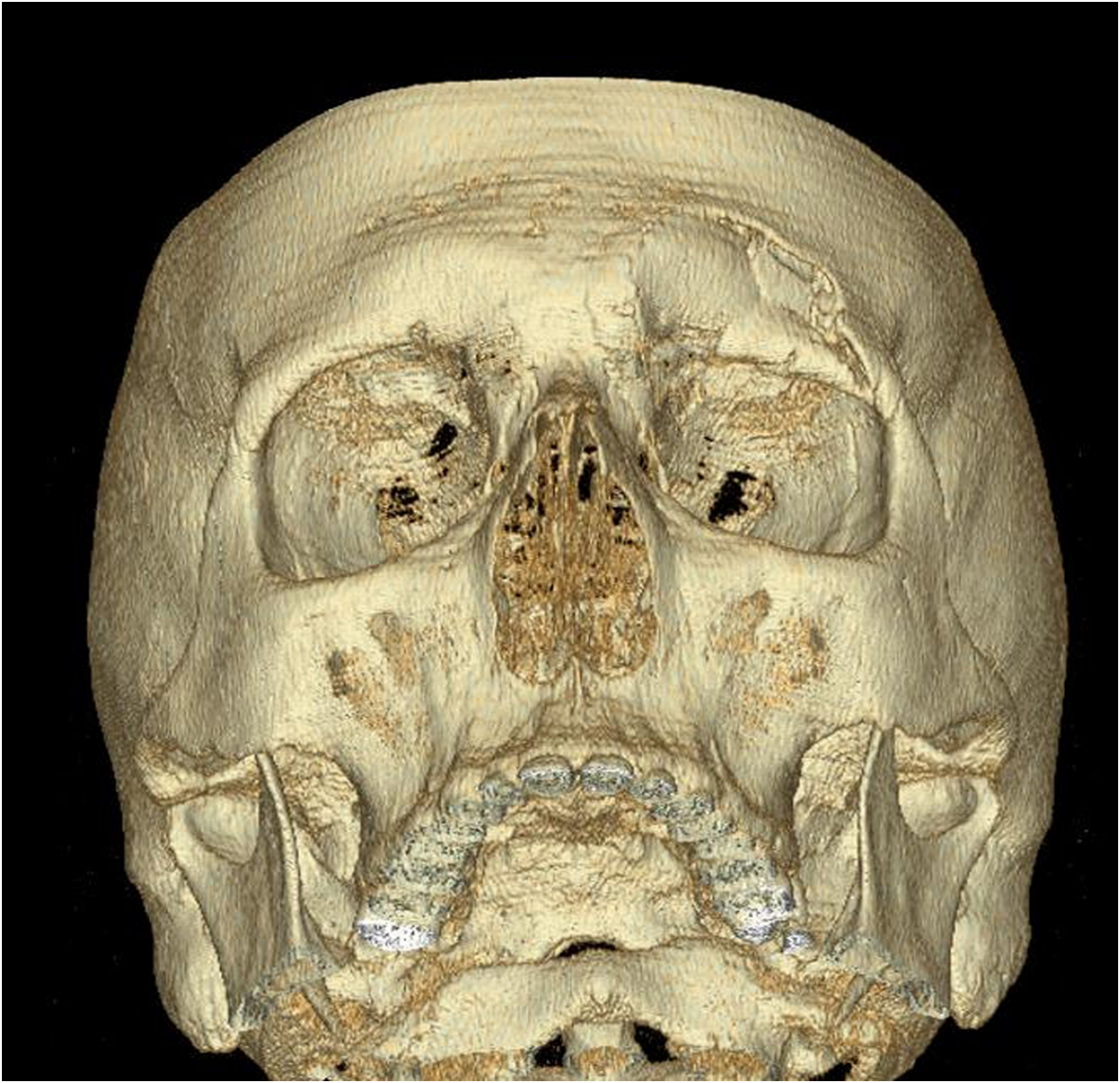
**High-impact trauma to the supraorbital region.** Impact caused by aberrant, high velocity wooden wedge (cutting wood logs with a circular saw) demonstrating comminuted fracture of the supraorbital arch and anterior orbital roof.

An explanation is the different architecture, where the supraorbital region is built by two outward bulging chords connected by struts. In civil engineering this design is used in arch dams or arch bridges known for their durability. Isolated limited supraorbital fractures are clinically rarely seen, this may be due to the fact that the impact was too low, that they could be easily overseen or that fist punches are more directed to the central face. According to literature [1,2] and our own experience, most fractures of the forehead are related to high-impact trauma and present as comminuted fractures (Figure 5).

Regarding the biomechanical cause for supraorbital blow-in fractures, which present downward displaced bony fragments of the superior orbital wall, our study gives some hints on their creation. As seen in Figure 4, stresses are transmitted to the orbital roof and it seems conceivable that a higher impact and/or different impact angles could cause such fractures. Future studies could show if this conception could be appropriate.

## Conclusions

This investigation showed that the supraorbital region can withstand higher forces than the midface due to the different bony architecture. Fist blows to that area may cause only limited fractures or stay below the critical yield borders for bony failure. To achieve extensive trauma as seen in clinical routine, higher impacts seem to be necessary. Furthermore this simulation showed that the orbital roof is affected by trauma of the supraorbital area, too. Higher forces could therefore explain the generation of orbital blow-in fractures. FEA allows the simulation of trauma and its consequences. The authors’ setup to develop a realistic, highly detailed skull model which represents individual mechanical values of each single element seems an appropriate way for this kind of investigation and will be used for further studies in the future.

## Consent

Written informed consent was obtained from the patient for the publication of this report and any accompanying images.

## Competing interests

All authors declare that there are no financial or non-financial competing interests.

## Authors’ contributions

HHH initiated this investigation, developed the study protocol, and drafted the manuscript. AS was in charge for the FEM-model and the BoneMat® scripts. TH participated in the study protocol and manuscript draft and coordinated the FEM studies. All authors read and approved the final manuscript.

## References

[B1] Haug RH Van Sickels JE Jenkins WS Demographics and treatment options for orbital roof fractures Oral Surg Oral Med Oral Pathol Oral Radiol Endod 2002 93 238 245 10.1067/moe.2002.120975 11925530

[B2] McGuire TP Gomes PP Clokie CML Sandor GKB Fractures of the supraorbital rim: principles and management J Can Dent Assoc 2006 72 537 540 16884645

[B3] Sullivan WG Displaced orbital roof fractures: presentation and treatment Plast Reconstr Surg 1991 87 657 661 10.1097/00006534-199104000-00008 2008463

[B4] Schaller A Voigt C Huempfner-Hierl H Hemprich A Hierl T Transient finite element analysis of a traumatic fracture of the zygomatic bone caused by a head collision Int J Oral Maxillofac Surg 2012 41 66 73 10.1016/j.ijom.2011.09.004 21996084

[B5] Schaller A Huempfner-Hierl H Hemprich A Hierl T Biomechanical mechanisms of orbital wall fractures – a transient finite element analysis J Craniomaxillofac Surg 2013 41 710 717 10.1016/j.jcms.2012.02.008 22417768

[B6] Vajgel A Carmargo IB Willmersdorf RB Menezes De Melo T Filho JRL De Holanda Vasconcellos RJ Comparative finite element analysis of the biomechanical stability of 2.0 fixation plates in atrophic mandibular fractures J Oral Maxillofac Surg 2013 71 335 342 10.1016/j.joms.2012.09.019 23351762

[B7] Parascandolo S Spinzia A Parascandolo S Piombino P Califano L Two load sharing plates fixation in mandibular condylar fractures: biomechanical basis J Craniomaxillofac Surg 2010 38 385 390 10.1016/j.jcms.2009.10.014 19944616

[B8] Waterhouse N Lyne J Urdang M Garey L An investigation into the mechanism of orbital blowout fractures Br J Plast Surg 1999 52 607 612 10.1054/bjps.1999.3194 10658130

[B9] Ahmad F Kirkpatrick WN Lyne J Urdang M Garey LJ Waterhouse N Strain gauge biomechanical evaluation of forces in orbital floor fractures Br J Plast Surg 2003 56 3 9 10.1016/S0007-1226(02)00467-8 12706141

[B10] Nagasao T Miyamoto J Nagasao M Ogata H Kaneko T Tamaki T Nakajima T The effect of striking angle on the buckling mechanism in blowout fracture Plast Reconstr Surg 2006 117 2373 2380 10.1097/01.prs.0000218792.70483.1f 16772944

[B11] Nagasao T Miyamoto J Shimizu Y Jiang H Nakajima T What happens between pure hydraulic and buckling mechanisms of blowout fractures? J Craniomaxillofac Surg 2010 38 306 313 10.1016/j.jcms.2009.09.001 19880325

[B12] Taddei F Pancanti A Viceconti M An improved method for the automatic mapping of computed tomography numbers onto finite element models Med Eng Phys 2004 26 61 69 10.1016/S1350-4533(03)00138-3 14644599

[B13] Szwedowski TD Whyne CM Fialkov JA Toward characterization of craniofacial biomechanics J Craniofac Surg 2010 21 202 207 10.1097/SCS.0b013e3181c50f64 20098185

[B14] Taylor WR Roland E Ploeg H Hertig D Klabunde R Warner MD Hobatho MC Rakotomanana L Clift SE Determination of orthotropic bone elastic constants using FEA and modal analysis J Biomech 2002 35 767 773 10.1016/S0021-9290(02)00022-2 12020996

[B15] Morgan EF Bayraktar HH Keaveny TM Trabecular bone modulus-density relationships depend on anatomic site J Biomech 2003 36 897 904 10.1016/S0021-9290(03)00071-X 12757797

[B16] Scholz R Hoffmann F Von Sachsen S Drossel WG Klöhn C Voigt C Validation of density-elasticity relationships for finite element modelling of human pelvic bone by modal analysis J Biomech 2013 46 2667 2673 10.1016/j.jbiomech.2013.07.045 24001928

[B17] Huiskes R Finite element analysis of acetabular reconstruction Acta Orthop 1987 58 620 625 10.3109/17453678709146499 3442206

[B18] Dechow PC Nail GA Schwartz-Dabney CL Ashman RB Elastic properties of human supraorbital and mandibular bone Am J Phys Anthropol 1993 90 291 306 10.1002/ajpa.1330900304 8460653

[B19] Whiting WC Gregor RJ Finerman GA Kinematic analysis of human upper extremity movements in boxing Am J Sports Med 1988 16 130 136 10.1177/036354658801600207 3377096

[B20] Tensi HM Gese H Ascherl R Non-linear three-dimensional finite element analysis of a cementless hip endoprosthesis Proc Inst Mech Eng H 1989 203 215 222 2701959 10.1243/PIME_PROC_1989_203_042_01

[B21] Gautam P Valiathan A Adhikari R Maxillary protraction with and without maxillary expansion: a finite element analysis of suturale stresses Am J Orthod Dentofacial Orthop 2009 136 361 366 10.1016/j.ajodo.2008.02.021 19732670

[B22] Yoganandan N Pintar FA Sances A Walsh PA Ewing CL Thomas DJ Snyder RG Biomechanics of skull fracture J Neurotrauma 1995 12 659 668 10.1089/neu.1995.12.659 8683617

[B23] Nahum AM The biomechanics of facial bone fractures Laryngoscope 1975 85 140 156 10.1288/00005537-197501000-00011 1113592

